# Bacterial biomarkers capable of identifying recurrence or metastasis carry disease severity information for lung cancer

**DOI:** 10.3389/fmicb.2022.1007831

**Published:** 2022-09-16

**Authors:** Xuelian Yuan, Zhina Wang, Changjun Li, Kebo Lv, Geng Tian, Min Tang, Lei Ji, Jialiang Yang

**Affiliations:** ^1^School of Mathematical Sciences, Ocean University of China, Qingdao, China; ^2^Department of Respiratory and Critical Care, Emergency General Hospital, Beijing, China; ^3^Geneis Beijing Co., Ltd., Beijing, China; ^4^Qingdao Geneis Institute of Big Data Mining and Precision Medicine, Qingdao, China; ^5^School of Life Sciences, Jiangsu University, Zhenjiang, Jiangsu, China; ^6^Chifeng Municipal Hospital, Chifeng, China

**Keywords:** lung cancer, recurrence or metastasis, bacterial community, random forest, survival

## Abstract

**Background:**

Local recurrence and distant metastasis are the main causes of death in patients with lung cancer. Multiple studies have described the recurrence or metastasis of lung cancer at the genetic level. However, association between the microbiome of lung cancer tissue and recurrence or metastasis remains to be discovered. Here, we aimed to identify the bacterial biomarkers capable of distinguishing patients with lung cancer from recurrence or metastasis, and how it related to the severity of patients with lung cancer.

**Methods:**

We applied microbiome pipeline to bacterial communities of 134 non-recurrence and non-metastasis (non-RM) and 174 recurrence or metastasis (RM) samples downloaded from The Cancer Genome Atlas (TCGA). Co-occurrence network was built to explore the bacterial interactions in lung cancer tissue of RM and non-RM. Finally, the Kaplan–Meier survival analysis was used to evaluate the association between bacterial biomarkers and patient survival.

**Results:**

Compared with non-RM, the bacterial community of RM had lower richness and higher Bray–Curtis dissimilarity index. Interestingly, the co-occurrence network of non-RM was more complex than RM. The top 500 genera in relative abundance obtained an area under the curve (AUC) of 0.72 when discriminating between RM and non-RM. There were significant differences in the relative abundances of *Acidovorax*, *Clostridioides, Succinimonas,* and *Shewanella*, and so on between RM and non-RM. These biomarkers played a role in predicting the survival of lung cancer patients and were significantly associated with lung cancer stage.

**Conclusion:**

This study provides the first evidence for the prediction of lung cancer recurrence or metastasis by bacteria in lung cancer tissue. Our results highlights that bacterial biomarkers that distinguish RM and non-RM are also associated with patient survival and disease severity.

## Introduction

Lung cancer is still the leading cause of cancer deaths worldwide. Local recurrence and distant metastasis are the primary causes of morbidity and mortality, and account for up to 95% of deaths related to lung cancer (Seyfried and Huysentruyt, 2013). Despite advances in therapeutic strategies, especially targeted therapy and immunotherapy, the prognosis remains poor because most patients have extensive metastases at diagnosis (Herbst et al., 2018; Liu et al., 2021). Clinically, a large number of patients with early-stage lung cancer relapse after surgery due to the neglected distant metastasis (Lu Y. et al., 2021). Thus, capturing the signal of metastasis in patients with early-stage lung cancer and continuously monitoring cancer progression after surgery is of great significance for reducing patient mortality.

Growing research has suggested that microbial communities influence the occurrence, progression, metastasis, and response to therapy of multiple cancers (Cullin et al., 2021; Yang M. et al., 2022). For example, studies have shown that *Fusobacterium nucleatum* may trigger cancer through multiple ways, and is related to cancer cell invasion and metastasis (Bullman et al., 2017). Recently, Bertocchi et al. found that intratumoral CRC-associated *Escherichia coli* could migrate to the liver following gut vascular barrier disruption and then prime the liver microenvironment to directly promote metastasis (Bertocchi et al., 2021). In addition, multiple studies have shown that enterotoxigenic *Bacteroides fragilis* could encode a toxin that ultimately induces chronic intestinal inflammation and tissue damage in colorectal cancer by targeting intestinal cells (Boleij et al., 2015; Cheng et al., 2020). However, the potential association between microbial communities of cancer tissue and lung cancer metastasis remains a knowledge gap.

A prominent reason for the high mortality rate of lung cancer is that it is initially asymptomatic and typically discovered at advanced stages (Nasim et al., 2019). Therefore, it is urgent to accurately identify the biomarkers in each stage of lung cancer and adjust the treatment measures for different stages (Yang et al., 2021). Zheng et al. identified 13 gut microbes as biomarkers with high accuracy in predicting early-stage lung cancer by 16 s rRNA sequencing analysis (Zheng et al., 2020). A survey of the gut and sputum microbiota of lung cancer patients at different stages by Lu et al. revealed that these two microbiomes were associated with distant metastasis and that microbial biomarkers across disease stages were largely shared (Lu H. et al., 2021). However, although the potential relationship between gut microbes and non-gut-related cancers is largely unraveled (Erdman and Poutahidis, 2015; Kwa et al., 2016; Zhao F. et al., 2021), the microbes at the original site of cancer development, the cancer tissue, deserve further exploration. The unclear mechanism of tissue microbiome in distant metastasis and lung cancer stage urgently needs to be investigated.

In this study, 174 samples of patients with recurrence or metastasis (RM) and 134 samples of patients without recurrence or metastasis (non-RM) were collected, and the tissue microbiome of all patients with lung cancer was characterized. The main objectives of this study were (1) to identify the bacterial biomarkers capable of discriminating between RM and non-RM, (2) to investigate the effect of smoking on RM and non-RM differential bacteria, and (3) to correlate bacterial biomarkers with survival and disease stage in lung cancer patients. Our study sheds light on the ability of tissue microbial markers of lung cancer to predict recurrence or metastasis and that these biomarkers are strongly associated with the survival and stage of patients with lung cancer.

## Materials and methods

### Patient cohorts and data preparation

Rob Knight’s team rechecked the microbial readings from 18,116 cancer tissue samples included 10,481 patients and 33 cancers in The Cancer Genome Atlas^1^ (TCGA; Poore et al., 2020). Of the 6.4′10^12^ sequencing readings in TCGA, 7.2% were classified as non-human, of which 35.2% were assigned to bacteria, archaea, or viruses; the sequencing readings were clustered into operational taxonomic units (OTUs) to the genus level by Kraken (Wood and Salzberg, 2014). Microbial sequencing technologies included whole-genome sequencing (WGS) and whole-transcriptome sequencing (RNA-seq). To obtain more tissue samples from lung cancer patients, we downloaded the microbial data obtained by RNA-seq in TCGA database, and obtained clinical indicators and patient information of all samples.

In total, we obtained 308 lung tissue samples from 298 patients with lung cancer. We divided the samples into two groups based on the presence of recurrence or metastasis within 3 years after the initial diagnosis of lung cancer. Specifically, we defined patient samples without recurrence or metastasis within 3 years as non-RM, and defined patient samples with recurrence or metastasis, or both recurrence and metastasis as RM. We obtained 174 RM samples and 134 non-RM samples. We also collected related important clinical indicators of the patients, such as age, gender, TNM stage, smoking history, etc. Specific information for all samples is provided in Table 1.

**Table 1:** Basic characteristics of study participants.

Characteristics	All (*n* = 308)	RM (*n* = 174)	Non-RM (*n* = 134)
Gender (F/M)	140/168	77/97	63/71
T stage (T1/T2/T3/T4/TX)	77/173/49/8/1	36/92/40/5/1	41/81/9/3/0
N stage (N0/N1/N2/N3/NX)	180/83/39/2/4	97/49/25/1/2	83/34/14/1/2
M stage (M0/M1/MX)	234/7/67	118/6/50	116/1/17
Stage (I/II/III/IV/Unknown)	140/98/60/7/3	64/63/40/6/1	76/35/20/1/2
Age (Mean ± SD)	65.44 ± 9.55	65.66 ± 9.27	65.15 ± 9.88
Histology (LUAD/LUSC)	173/135	114/60	59/75
Smoking history (Never/Reformed smoker≤15 years/Reformed smoker>15 years/Current smoker/Unknown)	30/136/57/71/14	20/68/35/43/8	10/68/22/28/6

LUAD, lung adenocarcinoma; LUSC, lung squamous cell carcinoma.

### Network analyses and keystone taxa

We performed network analysis to assess microbiome complexity and identify potential keystone genera for RM and non-RM. We used Spearman’s rank correlation to assess the association among genera. We used the *Hmisc* package for calculating correlation coefficients and *p* values. Correlation coefficients greater than 0.7 with a corresponding *p*-value less than 0.001 were considered statistically significant. Eligible correlations are used to generate the networks. The undirected networks were explored and visualized with the interactive platform Gephi (Bastian et al., 2009), using the *Fruchterman-Reingold* layout. Some important topological parameters and node scores of the resulting network are obtained through Gephi (Newman, 2006). In our networks, nodes represented the genera, and the edges represented Spearman’s rank correlations. The average degree is the number of edges on each node. Path length and diameter, respectively, represent the nearest distance and the largest distance between two nodes in a network. Clustering coefficient indicates the extent a node is connected to its neighbors. We used high degree to statistically identify the keystone taxa (Banerjee et al., 2019).

### Machine-learning classification model and biomarkers identification

We used the microbiome at the genus level as a feature to predict the possibility of recurrence or metastasis of patients in the future. We labeled the patients of RM as “0,” and the patients of non-RM as “1.” Thus, this problem can be considered a binary classification task. We selected Random Forest (RF) to complete our classification task. RF is used for classification purposes and it had a good performance in recent years. This model was implemented by Python’s Sklearn module. We estimated the performance of the classification algorithms using the 5-fold cross-validation (5-fold-cv) procedure. We evaluated the predicted goodness at each abundance level in steps of one hundred. The performance of the classification algorithm was estimated by averaging the area under the curve (AUC) in the 5 test datasets. To ensure comparability, the division of the datasets on each abundance was consistent.

We calculated the variable importance of the top 100 bacteria in relative abundance for identifying RM and non-RM using the Random Forest algorithm. We identified 15 bacterial biomarkers that best discriminated between RM and non-RM based on two variable importance metrics from Random Forest, mean decrease accuracy (MDA) and mean decrease gini (MDGini). Further, Wilcoxon rank-sum test was used to compare the differences of these 15 biomarkers between RM and non-RM. *p*-value <0.05 was considered statistically significant.

### Validation of predictions on survival

From the 15 bacterial biomarkers that discriminate between RM and non-RM, we used the bacteria with the top 6 variables in importance to predict all samples into two groups, recurrence or metastasis (Pred_lable = RM) and without recurrence or metastasis (Pred_lable = non-RM), respectively. Then, overall survival time and status were used to evaluate the prognosis of lung cancer patients in the two groups. The survival curve was performed by using the Kaplan–Meier method and the log-rank test was used to compare the difference in survival probability with R package “survival.” *p*-value <0.05 was considered statistically significant.

### Analysis of tissue microbes in different stages of lung cancer

The TNM staging system was first proposed by the French Pierre Denoix between 1943 and 1952(Asare et al., 2019), and later the American Joint Committee on Cancer (AJCC) and the Union for International Cancer Control (UICC) gradually began to establish an international system. In 1968, the first edition of the ‘*TNM Classification of Malignant Tumors*’ manual was officially published. It has become the standard method for staging malignant tumors by clinicians and medical scientists.

In the TNM staging system: (1) T refers to the condition of the primary tumor. With the increase in tumor volume and the increase in the extent of adjacent tissue involvement, it is represented by T1 ~ T4 in turn. (2) N refers to the involvement of regional lymph nodes. When the lymph nodes are not involved, it is indicated by N0. With the increase in the degree and scope of lymph node involvement, it is represented by N1 ~ N3 in turn. (3) M refers to distant metastasis (usually blood duct metastasis), M0 is used for those without distant metastasis, and M1 is used for those with distant metastasis. On this basis, use the combination of the three indicators of TNM to draw a specific stage. To investigate changes in bacterial composition at different stages of lung cancer, we compared the relative abundances of 15 bacterial biomarkers capable of distinguishing metastatic and non-metastatic between different stages. Wilcoxon rank-sum test was used to compare the differences between groups. Besides, taking the T stage as an example, we constructed five-fold cross-validation random forest models with features from the combinations of three bacterial biomarkers (*Dickeya*, *Lactococcus*, and *Pseudogulbenkiania*) to validate the performance of bacterial biomarkers in predicting the tumor stage.

### Identification of the patient’s smoking history

Based on the smoking history information of patients provided by TCGA, we divided all patients into four groups: smoking history >15 years, smoking history ≤15 years, current smokers, and unknown. Among them, the smoking age of current smoker is not clear, so we focused on comparing the relative abundance of bacterial biomarkers between the two groups of samples with a smoking history of more than 15 years and less than 15 years. Wilcoxon rank-sum test was used to compare the differences between groups.

### Statistical analysis

All analyses were implemented with R version 4.1.3^2^ and its appropriate packages. Principal coordinate analysis (PCoA) was performed with R package ‘ape’ based on the Bray-Curtis distance matrix. The Shannon index and Bray–Curtis dissimilarity index were calculated by using the R package “vegan.” Non-metric multidimensional scaling (NMDS) was performed with the R package “vegan.” Comparison between groups was conducted utilizing Wilcoxon rank-sum test. *p*-value <0.05 was set as the threshold.

## Results

### Characteristics of the lung cancer datasets in meta-analysis

A total of 308 lung tissue samples from 298 patients with lung cancer were obtained. We determined the recurrence or metastasis of patients based on the follow-up information provided by TCGA. Detailedly, we defined patients without recurrence or metastasis within three years after the initial diagnosis of lung cancer as non-RM samples, and patients with recurrence, metastasis, and simultaneous recurrence and metastasis within 3 years as RM samples. The demographics and clinical characteristics are provided in Table 1.

### Bacterial profile of the lung cancer microbiome is dominated by proteobacteria

**Figure 1:**
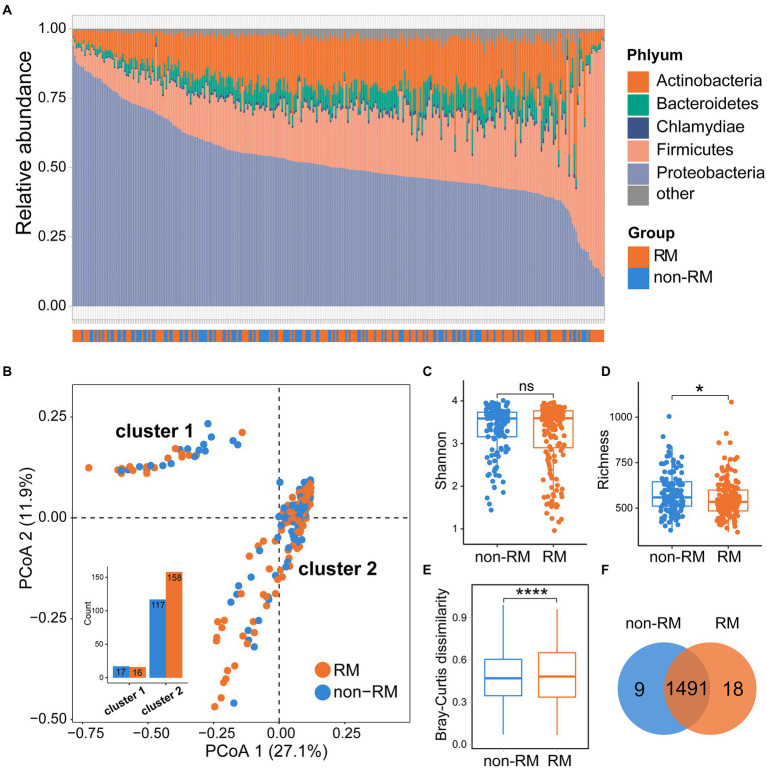
Bacterial community structures of RM and non-RM patients with lung cancer. **(A)** Bacteria composition at phylum level for all samples; **(B)** The PCoA plot, on the genus level, colored by group as in panel; Comparisons of **(C)** Shannon index and **(D)** richness of bacterial communities between RM and non-RM; **(E)** Bray–Curtis dissimilarity measures, on the genus level, based for all pairs of samples; **(F)** Common and unique genera between RM and non-RM. Wilcoxon test, * *p* < 0.05, **** *p* < 0.0001.

Previous microbial studies of lung cancer have shown that bacterial composition of cancerous lungs shifts compared to non-cancerous lungs (Huang et al., 2011); however, these compositional changes have not been examined in distant metastatic lung cancer. To obtain a comprehensive characteristic of the bacterial community of metastatic lung cancer, we thoroughly compared the bacterial compositions of RM and non-RM. As shown in Figure 1, Proteobacteria dominated the tissue microbiome of lung cancer with an average relative abundance of 52.3%, followed by Firmicutes (21.8%) and Actinobacteria (16.0%). Importantly, Proteobacteria was generally more dominant in non-RM (Wilcoxon *p* = 0.041), indicating that this is a recurrent phenomenon in lung cancer (Woerner et al., 2022).

### Bacterial composition carry information on recurrence or metastasis in lung cancer

We next computed the firth two principal coordinates based on the Bray–Curtis dissimilarity and the PCoA plot showed two distinct clusters (Figure 1B). The two groups (RM and non-RM) were not randomly dispersed between the two clusters. Instead, enrichment was observed in specific clusters for certain groups, providing further evidence that the bacterial composition may carry RM/non-RM information in lung cancer. Then, we examined the alpha diversity (Shannon) and richness of the microbiome within samples of RM and non-RM. Specifically, there was no significant difference in the Shannon index between RM and non-RM; however, we observed a significant increase in richness in non-RM as compared to RM tissue (Figures 1C,D). Further, we calculated the Bray–Curtis dissimilarity for each pair of samples to measure how different each pair is regarding bacterial composition. Non-RM samples were far more similar to one another than to the RM samples (Wilcoxon *p* < 0.0001; Figure 1E). We detected 1,509 and 1,500 genera in non-RM and RM, respectively, indicating that the vast majority of genera were shared in lung cancer tissues regardless of recurrence or metastasis (Figure 1F).

### Co-occurrence networks and keystone taxa of RM and non-RM

**Figure 2:**
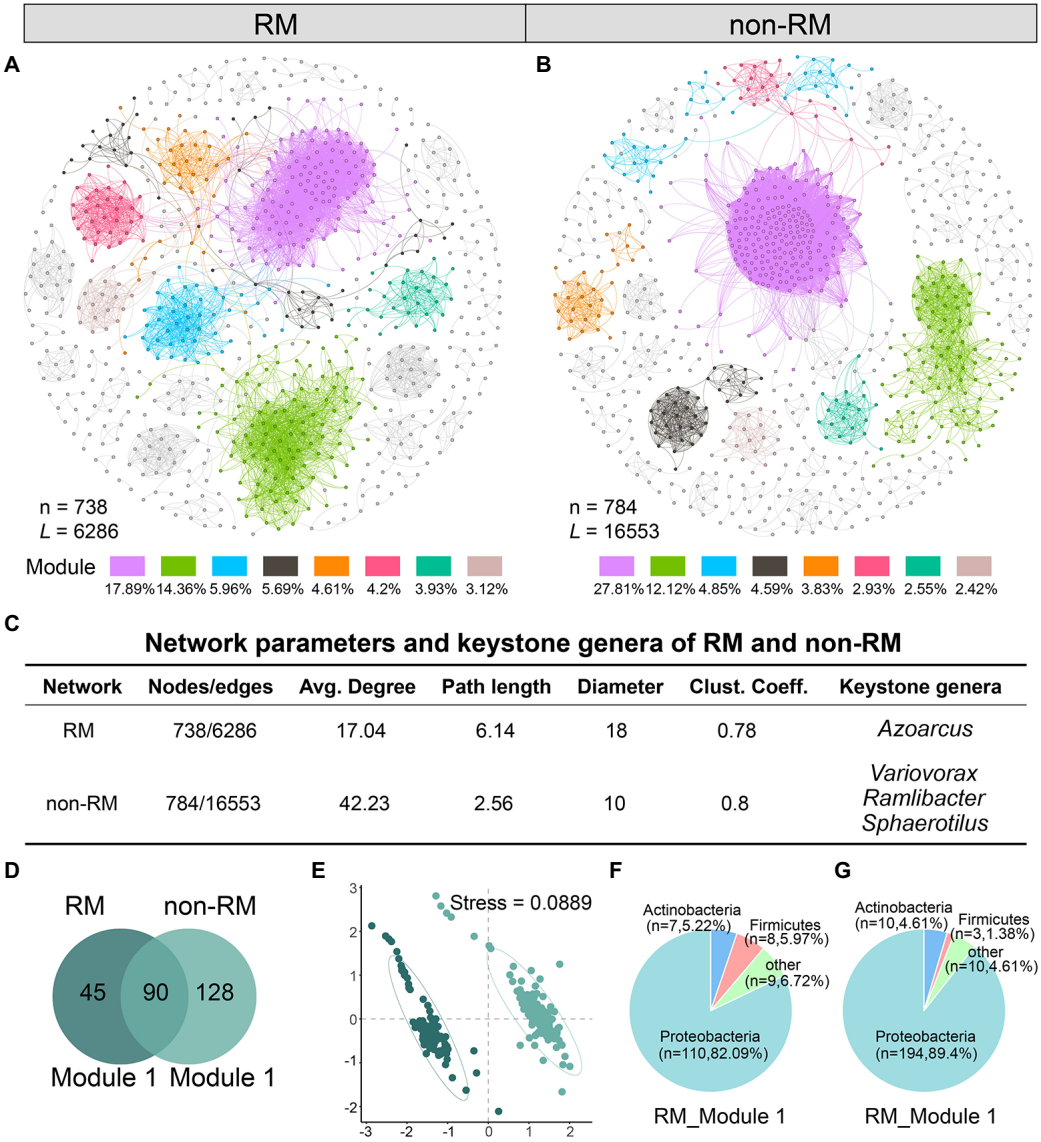
Network analysis reveals distinct bacterial community interaction patterns between RM and non-RM. Network of co-occurring bacteria of **(A)** RM and **(B)** non-RM. Only Spearman’s correlation coefficient (*r* > 0.7 significant at *p* < 0.001) is shown. The nodes are colored according to module. The percentage indicates the ratio of the number of nodes in the module to the total number; **(C)** Network parameters and the potential keystone genera of RM and non-RM. Average degree is the number of edges on each node. Path length and diameter, respectively, represent the nearest distance and the largest distance between two nodes in a network. Clustering coefficient indicates the extent a node is connected to its neighbors; **(D)** Common and unique genera in the largest modules of RM and non-RM; **(E)** NMDS analysis shows significant differences in the largest modules of RM and non-RM; Phylum-level bacterial composition in the largest modules of **(F)** RM and **(G)** non-RM.

We know that bacterial composition in the tissues of lung cancer patients with and without metastasis is different; however, the interaction pattern of bacterial communities in lung cancer tissues has not been disclosed. To reveal the underlying patterns, based on genus pairs with significant positive correlations screened by thresholds, we mapped co-occurrence networks for RM and non-RM, respectively (Figures 2A,B). Network complexity varied considerably between the two groups. Specifically, compared to non-RM, microbial communities in RM had a less complex network with fewer edges (6286), fewer nodes (738), a lower average degree (17.04), and a lower average clustering coefficient (0.78, Figure 2C). In the RM, the keystone genera we detected was *Azoarcus*, while in the non-RM, it was *Variovorax*, *Ramlibacter*, and *Sphaerotilus*. Although limited studies have directly linked these genera to lung cancer metastasis, the difference in keystone certainly implies a divergence in bacterial interactions between RM and non-RM.

Further, we drilled down into the largest module in the network, i.e., with the most nodes, which we called Module 1 in this study. Module1 of RM and non-RM contained 135 and 218 genera, respectively, of which 90 were shared (Figure 2D). NMDS analysis showed that the bacterial composition in Module 1 of the two groups was significantly different (Figure 2E; stress = 0.0889). Detailedly, Proteobacteria were the core phylum in these two modules, accounting for 82.1 and 89.4%, respectively (Figures 2F,G), further indicating that Proteobacteria dominated the lung cancer tissue bacterial community.

### Bacterial biomarkers for differentiating RM and non-RM are associated with patient outcomes in lung cancer

**Figure 3:**
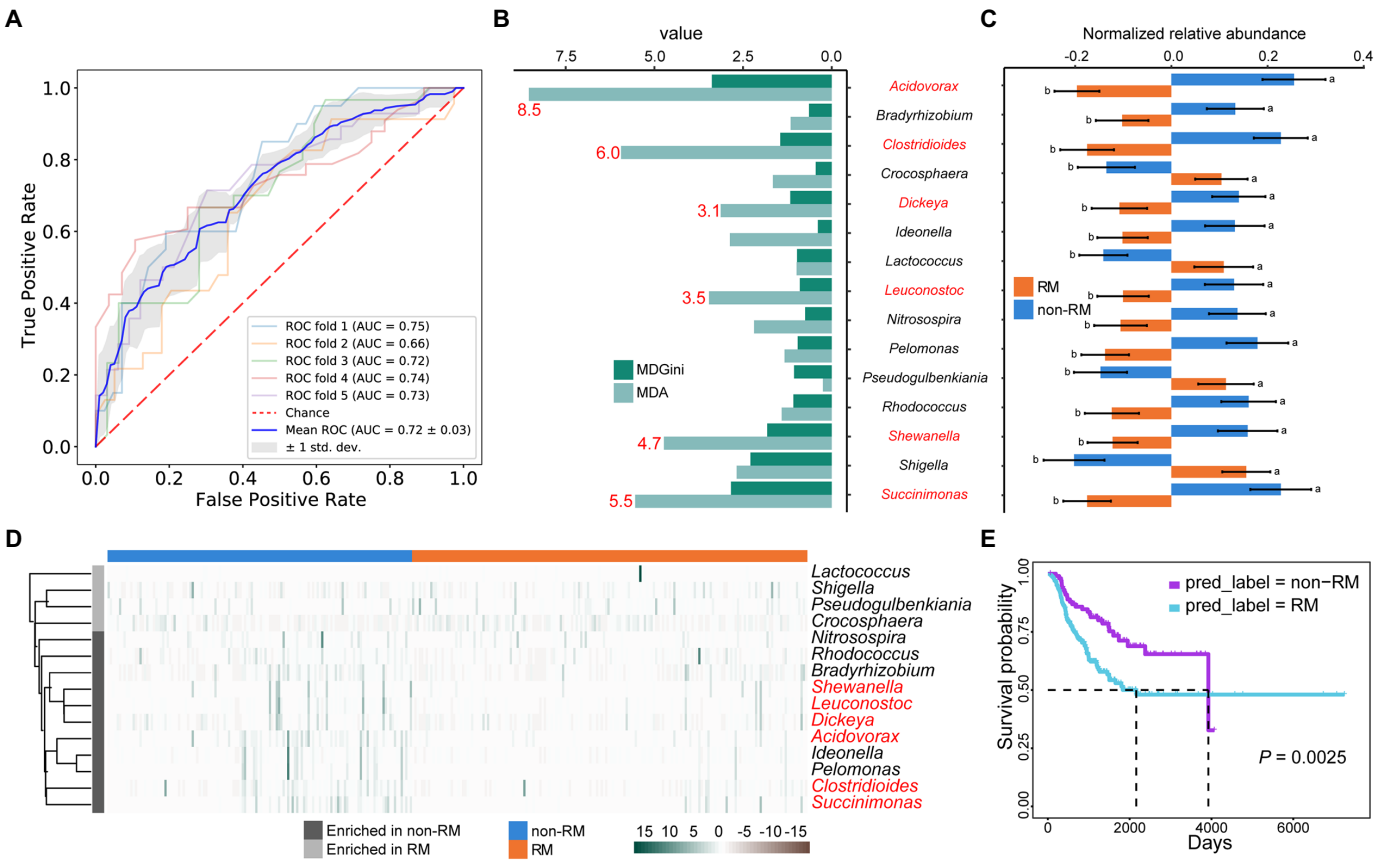
Metastasis and survival in lung cancer patients share some bacterial biomarkers. **(A)** Fivefold cross-validation random forest models with feature from top 500 genera in relative abundance; **(B)** The top 15 genera of variable importance predicted by random forest. The top 6 genera of MDA are shown in red; **(C)** The relative abundances of these 15 genera were significantly different between RM and non-RM; **(D)** Heatmap shows the enrichment of these 15 genera in RM and non-RM; **(E)** Kaplan–Meier survival curve shows that non-RM showed a significant overall survival benefit as compared with RM.

Given the observed differences in bacterial content between RM and non-RM (Figures 2A,B,3), we reasoned that bacteria might be able to classify recurrence or metastasis of patients with lung cancer. To this end, we constructed a machine-learning classifier to identify recurrence or metastasis from tissue bacteria. The top 500 genera in relative abundance were chosen as a compromise between reduced resolution with more genera and decreased representation of bacterial community with fewer genera. The average AUC of the classifier using bacterial content reached 0.72 (Figure 3A). The performance of our machine-learning classifier provides evidence that bacterial composition contains a signal that tracks recurrence or metastasis.

In addition to providing an algorithm to assign classes (RM/non-RM in our case) based on features (relative abundance of bacterial genera). Random Forest also assigns variable importance to each categorical feature. Based on the MDA value, we identified the top 15 genera with variable importance and significant differences between RM and non-RM (Figures 3B,C). *Acidovorax* has been reported to develop as a panel of sputum biomarkers that could diagnose lung squamous cell carcinoma (Leng et al., 2021). It is currently known that patients with lung cancer are at high risk of developing *Clostridium difficile* infection (CDI) due to continued chemotherapy, prolonged hospital stay, and general debility (Hwang et al., 2013). However, we detected a reduced relative abundance of *Clostridium* in recurrence or metastasis lung cancer patients, suggesting that its mechanism in recurrence or metastasis remains to be elucidated. Many other genera that contribute to discrimination between RM and non-RM (Figure 3D) are known to be associated with lung disease or lung cancer chemotherapy outcomes, e.g., *Leuconostoc* (Zhao Z. et al., 2021), *Shigella* (Zhang et al., 2018), *Rhodococcus* (Haramati and Jenny-Avital, 1998), and *Bradyrhizobium* (Jin et al., 2019).

Given the significance of predicting the prognosis of lung cancer patients and the relationship between bacteria and lung cancer patient survival demonstrated by multiple studies (Salazar et al., 2020; Tomita et al., 2020; Zhao Y. et al., 2021), we tried to correlate these bacterial biomarkers with patient survival. First, we selected the top 6 genera of variable importance as biomarkers (Figure 3D), then used these biomarkers to predict the recurrence or metastasis of all lung cancer patients, and finally performed survival analysis on the predicted two groups. As shown in Figure 3E, non-RM showed a significant overall survival benefit as compared with RM (*p* = 0.0025). Our results further prove the accuracy and clinical significance of the bacterial biomarkers we identified, as well as the fact that the patients with lung cancer recurrence or metastasis have reduced survival.

### Smoking history influences bacteria that distinguish recurrence or metastasis in lung cancer patients

Smoking is the greatest risk factor for lung cancer, up to 90% of lung cancers can be attributable to smoking (de Groot et al., 2018). Previous studies have demonstrated that nicotine-induced N2-neutrophils have a pro-metastatic role in lung cancer cell colonization (Tyagi et al., 2021). However, whether bacteria are mediators linking smoking and lung cancer recurrence or metastasis is still unknown.

**Figure 4:**
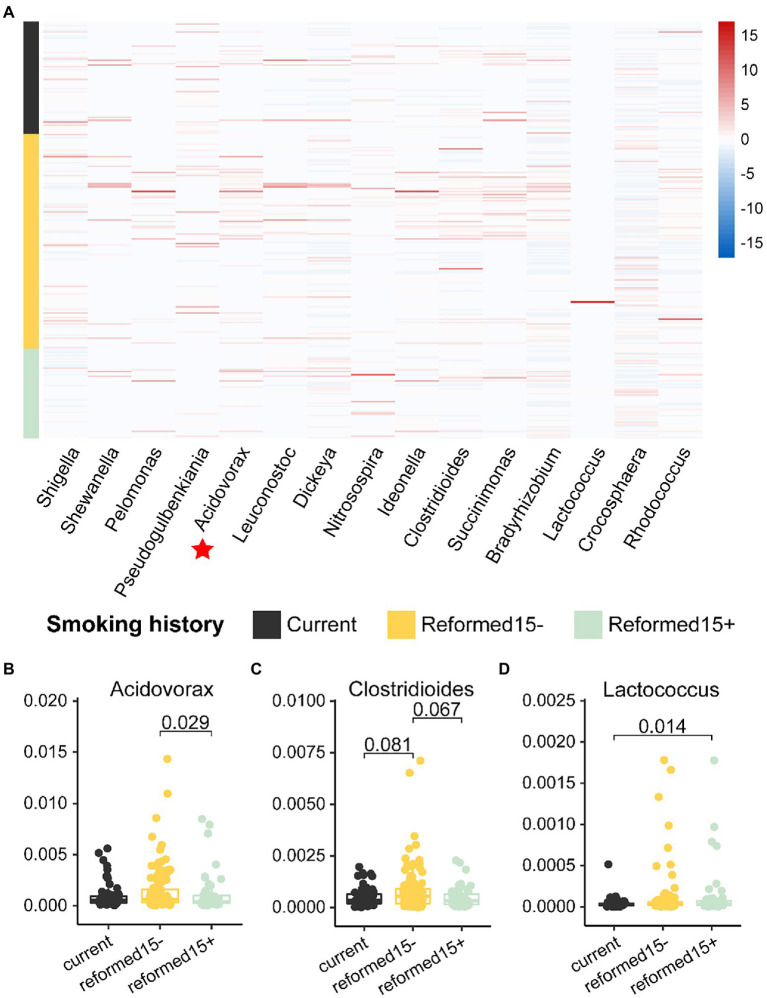
Relative abundances of bacterial biomarkers vary in patients with different smoking histories. **(A)** Heatmap shows the relative abundance of 15 bacterial biomarkers in patients with different smoking histories. The relative abundances of **(B)** *Acidovorax*, **(C)** *Clostridioides,* and **(D)** *Lactococcus* are significantly different among patients with different smoking histories.

Thus, we associated smoking history with recurrence or metastasis-related bacterial biomarkers (Figure 3B) in lung cancer patients. Coincidentally, we found that the relative abundance of most bacterial biomarkers was reduced in patients with a longer smoking history (Figure 4A). The relative abundances of the genera *Acidovorax*, *Clostridioides*, and *Lactococcus* varied with smoking history (Figures 4B–D). In particular, the relative abundance of the genus *Acidovorax* was significantly higher in patients with a smoking history of less than 15 years than in patients with a smoking history of more than 15 years (Figure 4B). Similarly, we also detected a reduced relative abundance of *Acidovorax* in RM compared to non-RM (Figure 3C). Naturally, we speculate that excessive smoking can cause changes in the content of certain bacteria, which, in turn, promotes the recurrence or metastasis of lung cancer patients.

### Bacterial biomarkers of disease stage and lung cancer recurrence or metastasis intersect

It has long been recognized that regional and metastatic cancers have a worse prognosis, and many cancers can be traced back to this gradual progression (Cserni et al., 2018). This has become the basis for cancer staging, including lung cancer. The Tumor-Node-Metastasis (TNM) system established by the Union for International Cancer Control (UICC) has become a worldwide means of describing the anatomical extent of cancer and determining its stage.

**Figure 5:**
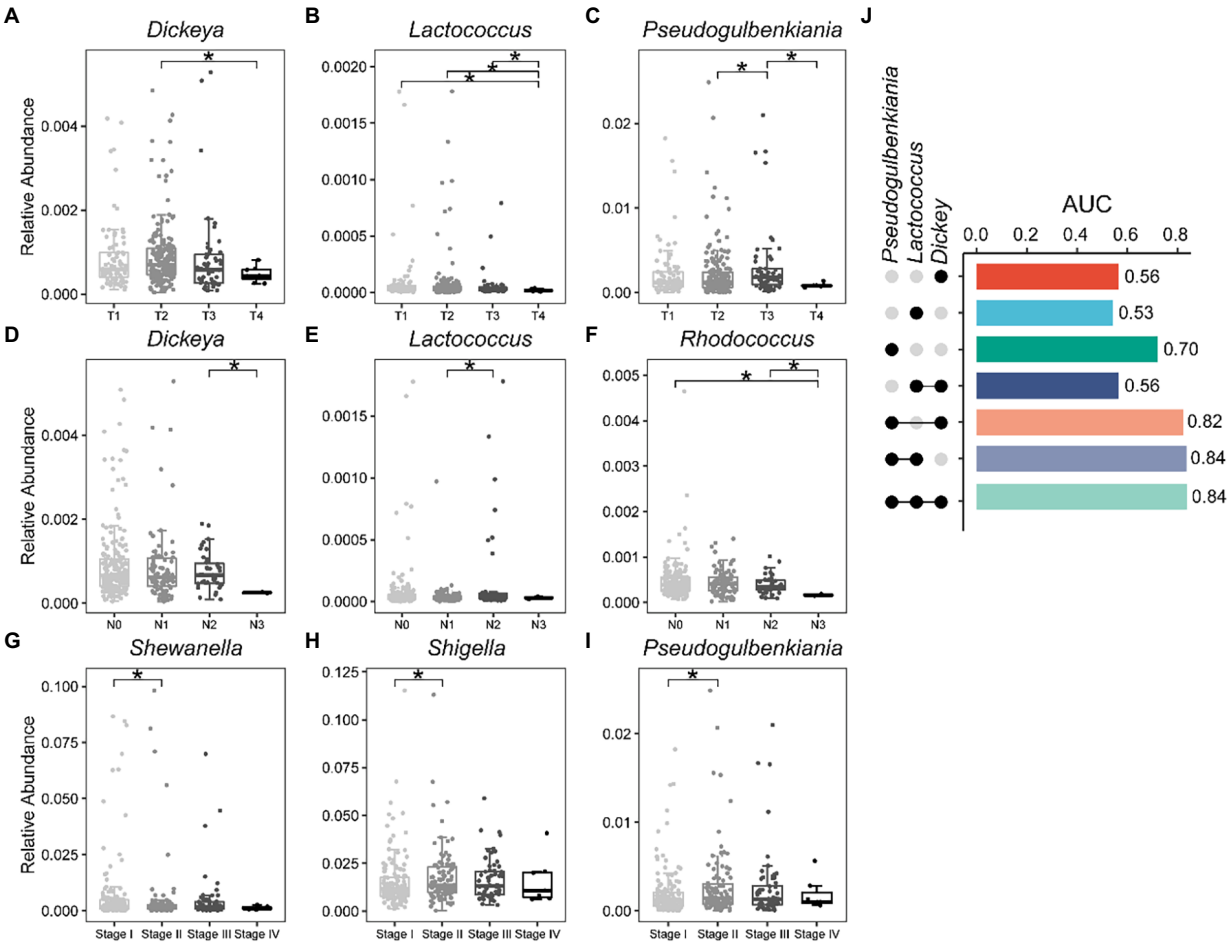
Bacterial biomarkers can identify disease stage in lung cancer patients. Relative abundance of specific genera varies significantly between different stages of lung cancer. **(A–C)** T stage; **(D–F)** N stage; **(G–I)** Stage; **(J)** Fivefold cross-validation random forest models with features from *Dickeya*, *Lactococcus,* and *Pseudogulbenkiania* to predict the T stage of lung cancer patients. Wilcoxon test, ^*^p < 0.05.

We have known that bacterial biomarkers that can distinguish recurrence or metastasis of lung cancer are related to patient survival (Figure 3E), and then we wondered whether these biomarkers also carry disease stage information. Interestingly, we found that the relative abundances of some of these 15 bacterial markers (Figure 2B) varied significantly between stages (Figures 5A–I). For example, the relative abundance of *Dickeya* was significantly lower in T4 patients compared to T2 patients (Figure 5A). *Rhodococcus* has the lowest relative abundance in N3 stage patients compared to other stages (Figure 5F). Then, taking the T stage as an example, we constructed five-fold cross-validation Random Forest models with features from these three biomarkers (Figures 5A–C). As expected, features from the combination of these three bacteria showed capabilities for identifying the T stage for patients with lung cancer (Figure 5J). The genera *Dickeya*, *Lactococcus*, and *Pseudogulbenkiania* displayed the strongest ability to identify the T stage with an average AUC of 0.84.

Wu and his colleagues found that mannan exopolysaccharides (EPS) produced by a subsp. of *Lactococcus lactis* affected the production of inflammatory cytokine (Wu et al., 2016). Similarly, we also detected a decrease in the relative abundance of *Lactococcus* in advanced patients (Figure 5B). In general, our results demonstrate that bacteria capable of discriminating recurrence or metastasis from lung cancer also carry disease stage information, thereby assisting clinicians and medical scientists in staging malignancies.

## Discussion

Genetic and environmental factors have long been recognized as contributors to cancer recurrence or metastasis (Bhujwalla et al., 2001; Rosell and Karachaliou, 2015; Song et al., 2020). Recently, histopathological images are also been found capable of predicting cancer recurrence or metastasis (Yang J. et al., 2022; Ye et al., 2022); however, little is known about the tissue microbiome that promotes cancer recurrence or metastasis. We demonstrate that recurrence or metastasis in lung cancer patients is associated with specific bacteria and that smoking significantly affects the relative abundance of these bacteria. In-depth, by building machine-learning classifiers, we found that six recurrence- or metastasis-distinguishing bacterial biomarkers (*Acidovorax*, *Clostridioides*, *Succinimonas*, *Shewanella*, *Leuconostoc*, and *Dickeya*) were associated with survival in lung cancer patients. Further, we verified that bacteria capable of discriminating recurrence or metastasis also carry information on tumor stage in lung cancer, and three genera, *Dickeya*, *Lactococcus*, and *Pseudogulbenkiania*, can accurately predict tumor T stage. Collectively, the above results support our proposal that smoking can lead to changes in the bacterial community in lung cancer tissue, which, in turn, affects tumor metastasis in patients, and the bacteria closely associated with recurrence or metastasis are inseparable from patient prognosis and tumor stage.

The number one risk factor for lung cancer development is tobacco exposure, which outweighs all other factors that lead to lung cancer (Bade and Dela Cruz, 2020). Tobacco smoke contains many potential carcinogens and bacterial products, which can induce epithelial cells to secrete inflammatory cytokines, cause barrier function impairment, and even alter the microbiome to influence lung carcinogenesis (Sapkota et al., 2010; Pauly and Paszkiewicz, 2011; Heijink et al., 2012; Checa et al., 2016). We observed significantly lower relative abundances of certain bacteria, such as *Acidovorax*, in patients with a smoking history of more than 15 years compared with patients with a smoking history of less than 15 years. Similarly, in a study of tumor (*n* = 143) and non-tumor adjacent tissues (*n* = 144), they observed a significant difference in the relative abundance of *Acidovorax* among smokers as compared to non-smokers (Greathouse et al., 2018). In addition, a study of non-malignant lung tissue showed that a greater abundance of *Acidovorax* was specifically found in the extracellular vesicles of smokers (Kim et al., 2017). Innovatively, we are the first to suggest that the relative abundance of *Acidovorax* is reduced in recurrence or metastasis patients compared to without recurrence or metastasis patients, echoing the reduced relative abundance of *Acidovorax* in patients with a longer smoking history. Nevertheless, future studies should mechanistically elucidate the role of *Acidovorax* between tobacco exposure and lung cancer metastasis.

Lung cancer patients face severe mortality even when detected in the early stages of cancer. Different from other types of cancers that are detected early and have obvious survival advantages, about 35–45% of patients with stage I lung cancer will die due to recurrence within 5 years even if the operation is successful (Molina et al., 2008; Zhao et al., 2017). We have verified that lung cancer patients with recurrence or metastasis are associated with lower survival rates. Further, our machine-learning classifier with features of *Acidovorax*, *Clostridioides*, *Succinimonas*, *Shewanella*, *Leuconostoc*, and *Dickeya* predicted recurrence or metastasis information in lung cancer patients, and patients predicted to be recurrence or metastasis had lower survival rates. Although the mechanism remains to be elucidated by more evidence, the current findings undoubtedly provide guidance for clinicians to preliminarily judge patient survival.

Our study showed that bacteria capable of distinguishing recurrence or metastasis can predict tumor stage in patients. In a study of 156 incident lung cancer cases and 156 individually matched controls, they found that species *Lactococcus lactis* was associated with decreased lung cancer risk (Shi et al., 2021). We also detected a decrease in the relative abundance of *Lactococcus* from the T1–T4 stages. *Dickeya*, *Lactococcus*, and *Pseudogulbenkiania* outperformed in predicting T4 and T1 stages in lung cancer patients.

The strength of our findings includes two accurately divided lung cancer cohorts with and without recurrence or metastasis within 3 years, the microbiome at the site of initial cancer development, and detailed follow-up information for nearly all patients. But our research still has some limitations. The distribution of samples in different stages is not uniform; for example, the number of samples in the T4 stage is much smaller than that in the T2 stage, we admit that this may skew the results. Although we comprehensively compared the tissue microbiome of patients without and those with recurrence or metastasis, the absence of healthy controls is a pity. Functional experiments are needed in the future to determine if and how bacteria influence the progression of lung cancer. Such experiments will reveal the potential of bacteria as biomarkers in lung cancer recurrence or metastasis and may provide treatment options for patients. Functional experiments to further provide treatment assistance for lung cancer patients is the focus of our future work.

## Conclusion

Through a comprehensive comparison of tissue microbes in recurrence or metastasis and without recurrence or metastasis lung cancer patients, we identified 15 bacterial biomarkers that differentiate between RM and non-RM lung cancer, with the relative abundance of most bacteria decreasing in recurrence or metastasis patients. Besides, six recurrence or metastasis-distinguishing bacterial biomarkers (*Acidovorax*, *Clostridioides*, *Succinimonas*, *Shewanella*, *Leuconostoc*, and *Dickeya*) were associated with survival in lung cancer patients. Further, we found that patients with longer smoking history were associated with lower abundances of these biomarkers, such as the genus *Acidovorax*. Finally, these bacterial biomarkers (*Dickeya*, *Lactococcus*, and *Pseudogulbenkiania*) accurately predicted the tumor T stage in lung cancer patients. We propose that smoking induces tissue microbial changes in lung cancer patients, which, in turn, promotes recurrence or metastasis in lung cancer patients, and the altered bacteria are associated with patient prognosis and tumor progression. With these results, we foresee a new avenue for mechanistic studies to address the role of microbes in the recurrence or metastasis of lung cancer patients, patient prognosis, and tissue tumor progression monitoring.

## Data availability statement

The original contributions presented in the study are included in the article/supplementary material, further inquiries can be directed to the corresponding authors.

## Ethics statement

Written informed consent was obtained from the individual(s) for the publication of any potentially identifiable images or data included in this article.

## Author contributions

JY, LJ, and CL conceived this study. XY and ZW performed the study and experiments. KL, GT, and MT collected the data. XY and LJ wrote the manuscript. All authors contributed to the interpretation of data and to the revision of the manuscript.

## Conflict of interest

GT, LJ, and JY were employed by Geneis Beijing Co., Ltd.

The remaining authors declare that the research was conducted in the absence of any commercial or financial relationships that could be construed as a potential conflict of interest.

## Publisher’s note

All claims expressed in this article are solely those of the authors and do not necessarily represent those of their affiliated organizations, or those of the publisher, the editors and the reviewers. Any product that may be evaluated in this article, or claim that may be made by its manufacturer, is not guaranteed or endorsed by the publisher.

## Footnotes

^1^ https://portal.gdc.cancer.gov

^2^ http://www.R-project.org
